# Adherence to guidelines across different specialties to prevent infections in patients undergoing immunosuppressive therapies

**DOI:** 10.1186/s12879-020-05082-8

**Published:** 2020-05-20

**Authors:** David R. Chadwick, Laila Sayeed, Matthew Rose, Emily Budd, Mo Mohammed, Sarah Harrison, Jaskiran Azad, Jamie Maddox

**Affiliations:** 1 Centre for Clinical Infection, James Cook University Hospital, Middlesbrough, TS4 3BW UK; 2 Department of Haematology, James Cook University Hospital, Middlesbrough, UK; 3 Undergraduate Department, James Cook University Hospital, Middlesbrough, UK; 4 Department of Dermatology, James Cook University Hospital, Middlesbrough, UK

**Keywords:** Infection, Immunosuppression, Biologic, Immunocompromised, Reactivation, Chemotherapy, Vaccine, Prophylaxis

## Abstract

**Background:**

Substantial numbers of patients are now receiving either immunosuppressive therapies or chemotherapy. There are significant risks in such patients of developing opportunistic infections or re-activation of latent infections, with higher associated morbidity and mortality. The aim of this quality improvement project was to determine how effective 5 different specialties were in assessing and mitigating risks of developing opportunistic infections or re-activation of latent infections in patients undergoing immunosuppressive therapies.

**Methods:**

This was a single centre audit where records of patients attending clinics providing immunosuppressive therapies were reviewed for the following: evidence of screening for blood-borne virus [BBV] infections, varicella and measles immunity, latent/active TB or hypogammaglobulinaemia, and whether appropriate vaccines had been advised or various infection risks discussed. These assessments were audited against both national and international guidelines, or a cross-specialty consensus guideline where specific recommendations were lacking. Two sub-populations were also analysed separately: patients receiving more potent immunosuppression and black and minority ethnic [BME] patients,.

**Results:**

For the 204 patients fulfilling the inclusion criteria, BBV, varicella/measles and latent TB screening was inconsistent, as was advice for vaccinations, with few areas complying with specialty or consensus guidelines. Less than 10% of patients in one specialty were tested for HIV. In BME patients screening for HIV [60%], measles [0%] and varicella [40%] immunity and latent [30%] or active [20%] TB was low. Only 38% of patients receiving potent immunosuppression received *Pneumocystis* prophylaxis, with 3 of 4 specialties providing less than 15% of patients in this category with prophylaxis.

**Conclusions:**

Compliance with guidelines to mitigate risks of infection from immunosuppressive therapies was either inconsistent or poor for most specialties. New approaches to highlight such risks and assist appropriate pre-immunosuppression screening are needed.

## Background

Increasing numbers of patients are now receiving either immunosuppressive therapies or chemotherapy for autoimmune disorders, organ transplants or cancers. There are well-established risks in such patients of developing opportunistic infections or re-activation of latent infections, often with substantial associated morbidity and mortality [1–7]. A number of specialist societies or agencies, both national and international, as well as drug manufacturers have issued guidelines which often include recommendations to screen patients for latent infection risks prior to commencing immunosuppressive therapy and provide vaccinations or chemoprophylaxis to prevent certain infections [8–19]. There is considerable variation between these guidelines in terms of screening and vaccination advice, even for use of the same immunosuppressive therapies. Moreover some do not address particular infection risks, with the result that clinicians may be uncertain as to which guidelines to follow or which infections to screen.

Infections resulting from immunosuppressive therapy or chemotherapy differ depending on the element of the immune system targeted and previous exposure of the patient to different pathogens. They can be broadly categorised into opportunistic infections or reactivation of latent infections. Latent infections which may reactivate include TB, varicella and hepatitis B and patients should be screened for such infections prior to immunosuppressive therapy. This is particularly important in those originating from countries or communities where such infections are endemic. Identification of latent infections or lack of immunity to such pathogens permits either treatment prior to immunosuppression, vaccination or higher vigilance for reactivation. It is also important to screen patients for undiagnosed HIV infection given the additional immunosuppression resulting from this infection and higher risk of opportunistic or other infections [18, 20]. Some opportunistic or other important infections can be prevented by providing either vaccines, such as influenza or pneumococcal [17], or chemoprophylaxis: for example cotrimoxazole to prevent *Pneumocystis jiroveci* pneumonia [PJP] in patients who receive potent immunosuppression [21]. As haematological malignancies and some immunosuppressant therapies have the potential to cause hypogammaglobulinaemia, which is an additional and potentially correctable risk factor for opportunistic infections, it is also important to screen selected patients for this prior to starting immunosuppressive therapy or chemotherapy.

Few studies or audits have attempted to assess how well guidelines are applied in a broad area of preventing infections in patients undergoing immunosuppressive therapy or chemotherapy; for example two recent studies of patients undergoing haematopoietic stem cell transplants showed poor compliance with vaccination guidelines [22, 23]. Given the heterogeneity of available guidelines we wanted to assess how well different specialties adhered to both specialist society guidelines and a ‘consensus guideline’, which we developed from a range of other guidelines, in terms of reducing the risk of infections.

## Methods

### Patient selection

This was a retrospective single-centre audit conducted in five specialties in a secondary/tertiary referral hospital in North-East England. Consecutive adult patients attending five outpatient departments in January and February 2018 were assessed to determine if they had received immunosuppressive therapy or chemotherapy at any point in the previous 3 years (January 2015 to December 2017), with a target of 50 patients reviewed for each specialty. The specialties assessed were Dermatology, Rheumatology, Gastroenterology, Nephrology and Haematology. Patients were defined as eligible for inclusion if they had received either chemotherapy for haematological malignancies or immunosuppressive drugs or biologics for inflammatory bowel disease (IBD), autoimmune disorders or solid organ transplants for at least 2 weeks, or one intravenous drug infusion. The non-biologics drugs defined for patient inclusion were prednisolone [20 mg daily or more], tacrolimus, mycophenolate mofetil (MMF), leflunomide, ciclosporin, cyclophosphamide, azathioprine (100 mg daily or more) or methotrexate [10 mg daily or more]. Patients who had received any biologic immunosuppressive drug alone were also included.

### Audit standards and outcomes

The following items of an infection prevention strategy were assessed in patients identified as eligible for inclusion.

1. Discussion with patient around general risks of infections and live vaccine risks.
2. Screening for active or latent tuberculosis [TB]: chest x-ray and interferon-γ release assay [IGRA].
3. Screening for hepatitis B, hepatitis C and HIV infection, including previous hepatitis B exposure and immunity. For HIV the documented offer of a test was accepted if a test had not been ordered.
4. Screening for varicella zoster virus [VZV] immunity: documented previous infection or VZV IgG test.
5. Screening for measles immunity: documented previous infection or IgG test
6. Screening for hypogammaglobulinaemia
7. Advice or provision of influenza, pneumococcal vaccines, or varicella or hepatitis B vaccines if the patient was non-immune.
8. Provision of PJP prophylaxis, only for patients receiving potent immunosuppression – see definition below.

Patients who had received prednisolone 20 mg or more for > 1 month, plus another immunosuppressive agent or two immunosuppressive agents in combination were defined as having had potent immunosuppressive therapy and therefore needing PJP prophylaxis. Outcomes were assessed from medical records - including drug charts and correspondence to General Practitioners - and from the electronic pathology results system [ICE – Clinisys]. For TB screening, latent infection testing was assumed to have occurred if an IGRA test had been ordered [this was the recommended hospital test] and screening for active infection if a chest x-ray had been ordered. Data sources were reviewed for two periods, first the period in the 3 months prior to the start of immunosuppressive therapy [all items], and second a period 5 years prior to the start of therapy [all aside from items 1]. CMV immunity was not assessed in the audit since all transplant recipients received pre-transplant screening at the regional transplant unit in Newcastle.

For each specialty, auditable standards from national and/or international guidelines, which may have covered some of these items, were chosen by the specialty lead. Since none of the specialty guidelines covered all of these items, a ‘consensus guideline’ was developed (Supplemental Table 1) using a number of other national guidelines [17–19, 21], and applied where such gaps existed to complete full auditable outcomes for each specialty. The specific guidelines used for each specialty are shown in Table 1. For each item, a target standard of 95% of patients achieving each outcome was set as a quality measure.

**Table 1:** Specialty and general guidelines used for audit outcomes

Specialty	Society or other organisation guideline	Infection prevention items recommended^a^	Reference
**Dermatology**	British Association of Dermatology [Methotrexate], 2016	2–5, 7, 8	[12]
	British Association of Dermatology [Biologics], 2017	2–5, 7, 8	[13]
**Gastroenterology**	European Cohn’s & Colitis Organisation [2014]	2, 7, 8	[8]
	British Society of Gastroenterology [2011]	2, 7, 8	[9]
**Haematology**	British Society of Haematology [DLB Lymphoma] 2016	3	[11]
**Nephrology**	British Transplant Society	2–5, 7, 8	[10]
**Rheumatology**	European League against Rheumatism [DMARDs and vaccines], 2011 & 2016	7	[14, 15]
	British Society of Rheumatology [DMARD & psoriatic arthropathy] 2017	7	[16]
**Other guidelines [for consensus guidelines]**	UK Green Book [vaccinations]	7	[17]
	NICE Blood-borne virus screening	3	[18, 19]

^a^Items: 1. Discussion with patient around general risks of infections; 2. Screening for active or latent tuberculosis [TB]; 3. Screening for Hepatitis B & C and HIV infection, including previous hepatitis B exposure and immunity; 4. Screening for varicella immunity; 5. Screening for measles immunity; 6. Screening for hypogammaglobulinaemia; 7. Recommendation of influenza, pneumococcal, +/− other indicated vaccines; 8. Provision of *P. jiroveci* prophylaxis

### Data collection and analysis

Data was collected in a standardised audit Proforma and transferred to a database. Medical data collected included demographics and underlying condition with drugs used and duration. Demographic data was presented descriptively, and comparison of proportions of patients receiving interventions between specialties and the combined other specialties was done by pair-wise chi-squared tests, if the global test was < 0.05. Two sub-analyses were performed, first amongst BME patients at higher risk of hepatitis B and HIV infection, TB infection or reactivation or being measles non-immune; and second in those treated with potent immunosuppressive therapies with reference to whether PJP prophylaxis was given.

## Results

Data from 204 patients fulfilling the eligibility criteria were obtained for the main audit. The characteristics of this population is shown in Table 2.

**Table 2:** Characteristics of the population studied

	Dermatology [*n* = 38]	Gastroenterolgy [*n* = 42]	Haematology [*n* = 30]	Nephrology [*n* = 45]	Rheumatology [*n* = 49]	Combined population [*n* = 204]
Age [median], years	46	43	59	58	57	54
Male gender, n [%]	21 [47]	22 [51]	15 [50]	20 [44]	20 [41]	98 [48]
Non-biologic ISD^a^, n [%]						
Prednisolone^b^	0 [0]	12 [29]	22 [73]	41 [91]	7 14]	82 [40]
Azathioprine	0 [0]	13 [33]	1 [3]	3 [7]	1 [2]	19 [9]
Methotrexate	3 [8]	2 [5]	2 [7]	0 [0]	14 [29]	21 [10]
MMF^c^	0 [0]	0 [0]	1 [3]	20 [44]	0 [0]	21 [10]
Cyclophosphamide	0 [0]	0 [0]	17 [57]	16 [36]	1 [2]	34 [17]
Tacrolimus	0 [0]	0 [0]	3 [10]	20 [44]	0 [0]	23 [11]
Biologic ISD, n [%]						
Infliximab	1 [3]	37 [88]	0 [0]	0 [0]	16 [33]	54 [26]
Adelumimab	24 [63]	0 [0]	1 [3]	0 [0]	0 [0]	15 [12]
Etanercept	0 [0]	0 [0]	0 [0]	0 [0]	2 [4]	2 [1]
Secukinumab	1 [3]	0 [0]	0 [0]	0 [0]	0 [0]	1 [0.5]
Abatacept	0 [0]	0 [0]	0 [0]	0 [0]	5 [10]	5 [2]
Ustekinumab	10 [26]	0 [0]	0 [0]	1 [2]	0 [0]	11 [5]
Rituximab	1 [3]	0 [0]	12 [40]	10 [22]	19 [39]	42 [21]
Tociliuzumab	0 [0]	0 [0]	0 [0]	0 [0]	8 [16]	8 [4]
Other ISD^d^, n [%]	0 [0]	6 [14]	16 [53]	1 [2]	0 [0]	23 [11]
Duration of therapy, n [%]						
Up to 4 weeks	0 [0]	0 [0]	0 [0]	2 [4]	5 [10]	7 [3]
4 weeks to 1 year	10 [26]	10 [24]	14 [47]	24 [53]	5 [10]	63 [31]
More than 1 year	28 [74]	32 [74]	16 [53]	19 [42]	39 [80]	134 [66]

^a^ISD: immunosuppressant drug; ^b^ Or other steroid drug; ^c^ Mycophenolate mofitil. ^d^ includes both biologic and non-biologic immunosuppressant drugs

Most patients in Dermatology were treated for psoriasis, in Gastroenterology for IBD, in Haematology for lymphoma, myeloma or various leukaemias, in Nephrology for vasculitis or renal transplant, and in Rheumatology for rheumatoid or psoriatic arthritis or ankylosing spondylitis. Less than 5% of patients [10 of 204] were from BME backgrounds. The most common non-biologic drugs used were prednisolone [40%] and cyclophosphamide [17%]. The most common biologics used were anti-TNF ones [41%] and rituximab [20%]. Most patients were taking immunosuppressant therapy for either longer than one year [66%] or between 1 and 12 months [31%].

**Fig. 1:**
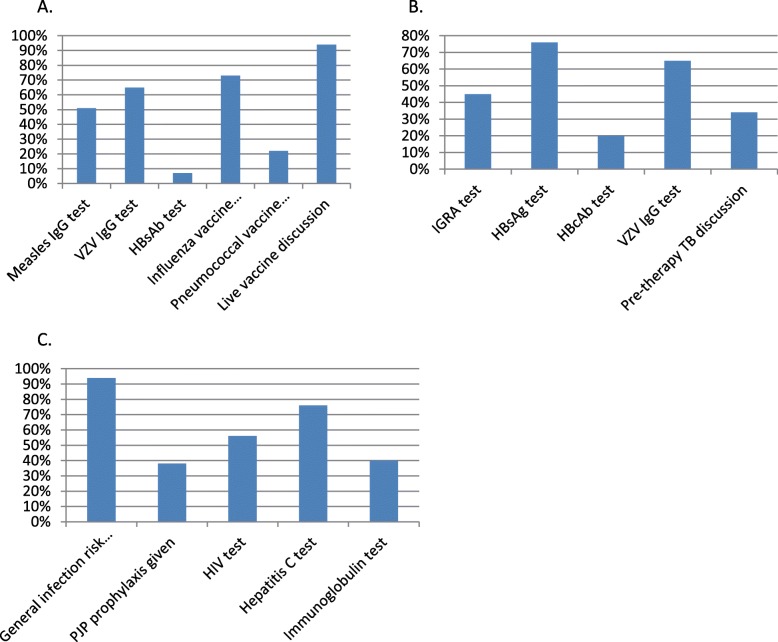
Audit outcomes categorised by intervention (percentage of patients having test or intervention). **a**. Interventions to prevent reactivation of infections. **b**. Screening or advice for vaccinations. **c**. Miscellaneous interventions to prevent infections or identify important chronic viral infections

The main audit outcomes are shown in Fig. 1 and Table 3, the latter of which shows whether items were screened up to 5 years before therapy, aside from item 1 [3 months].

**Table 3:** Audit outcomes for interventions to prevent infections

	Dermatology [*n* = 38]	Gastroenterolgy [*n* = 42]	Haematology [*n* = 30]	Nephrology [*n* = 45]	Rheumatology [*n* = 49]	Combined population [*n* = 204]
Tuberculosis screening, n[%]						
Pre-treatment discussion	37 [97]	40 [93]	3 [10]	1 [4]	27 [57]	70 [34]
Pre-treatment chest x-ray	33 [87]	37 [88]	25 [83]	40 [89]	37 [76]	172 [84]
IGRA^a^	30 [79]	34 [81]	1 [3]	4 [9]	23 [47]	90 [45]
Blood-borne virus screening, n[%]						
HIV	16 [42]	36 [84]	19 [63]	39 [87]	4 [8]	114 [56]
Hepatitis B [HBsAg]	35 [92]	34 [81]	19 [63]	38 [84]	30 [61]	156 [76]
Hepatitis B exposure [HBcAb]	0 [0]	4 [10]	12 [40]	20 [44]	4 [8]	40 [20]
Hepatitis B immunity [HBsAb]	0 [0]	3 [8]	3 [10]	8 [18]	1 [2]	15 [7]
Hepatitis C	35 [92	35 [83]	18 [60]	39 [87]	29 [59]	156 [76]
Other viral immunity screen, n[%]						
Varicella zoster immunity	37 [97]	26 [62]	6 [20]	23 [52]	33 [67]	133 [65]
Measles immunity	14 [38]	41 [98]	8 [27]	38 [84]	3 [6]	104 [51]
Hypogammaglobulinaemia screen, n[%]	4 [11]	1 [2]	23 [78]	21 [48]	32 [65]	81 [40]
Immunisation advice, n[%]						
Influenza	38 [100]	37 [88]	23 [78]	0 [0]	49 [100]	148 [73]
Pneumococcal^b^	0 [0]	37 [88]	0 [0]	0 [0]	7 [15]	44 [22]
Varicella zoster [if non-immune]	1 [25]	1 [20]	0 [0]	0 [0]	2 [50]	4 [30]
Hepatitis B [if non-immune]	0 [0]	0 [0]	0 [0]	1 [50]	0 [0]	1 [33]
Discussions on infection risk, n[%]						
General infection risks	38 [100]	38 [90]	22 [75]	45 [100]	48 [98]	191 [94]
Live vaccine risk	38 [100]	37 [88]	23 [78]	45 [100]	49 [100]	192 [94]
Provision of PJP prophylaxis^c^, n[%]	–	1/23 [4]	3/22 [14]	36/40 [90]	0/19 [0]	40/104 [38]

^a^IGRA: interferon-gamma release assay; ^b^ either PPV-23 or PCV-13; ^c^ in those receiving potent immunosuppression

There were only a few areas where individual specialties met the 95% standard and in the combined results no item achieved the 95% screening standard. TB screening items varied markedly across specialties, however only 45% of patients had an IGRA test, and 34% warned about the risk of TB developing on therapy. Screening for blood-borne viruses was poor, especially for HIV [56%], whilst 76% had hepatitis B and C tests. Testing for latent hepatitis B [by HBcAb] was also poor [20%].

In terms of advice to patients regarding which vaccines they should receive, influenza vaccine was advised in 73% of patients and pneumococcal vaccine in only 20%. Where patients were noted to lack immunity to VZV or hepatitis B, in only 30 and 33% of cases could advice to receive vaccination be seen, respectively. In contrast provision of advice to patients on general risks of infection whilst on immunosuppressive therapy, and the risk of receiving live vaccines was much better with 94% of patients receiving both types of advice.

In a sub-analysis of BME patients, 9 of 10 of whom were defined as receiving potent immunosuppressive therapy, although all had had a chest x-ray within 5 years of starting therapy, only 2 had undergone an x-ray and 3 an IGRA in the months prior to starting therapy. In this group screening within 3 months or 5 years was sub-optimal for HIV [4; 6 individuals], hepatitis B previous exposure - HBcAb [0; 10], hepatitis B immunity - HBsAb [10; 10] and measles immunity [0; 0], however somewhat better for Hepatitis B infection - HBsAg [6; 9], hepatitis C [7; 7] and VZV immunity [4; 8]. Only 6 of this group were recommended to have influenza vaccine and 5 a pneumococcal vaccine.

For patients receiving more potent immunosuppression only 40/104 [38%] patients were given PJP prophylaxis; the vast majority of these were attending the Nephrology clinic. In the overall population who had received potent immunosuppression, over the previous 5 years 67/104 [64%] were screened for HIV, 82/104 [79%] for both hepatitis B and C, whilst screening for varicella [65%] and measles [51%] immunity was suboptimal.

## Discussion

This audit was designed to assess whether patients undergoing immunosuppressive therapies had received appropriate screening, interventions and advice to address a broad range of potential infective complications. The lack of national guidelines covering all of these areas, and our observation that specialty guidelines often failed to address a number of areas where infectious risks exist, made it difficult for us to apply uniformly recognised standards. Likewise this has meant that specialists initiating immunosuppressive therapies are not always aware of all infection risks and able to anticipate them. Nonetheless by creating a ‘consensus’ guideline to ensure all areas of infection risk were applied to each specialty, we were able to apply a logical standard to patients’ management. There are a number of other guidelines or reports advising on such practice prior to giving immunosuppressive therapies in relation to organ transplantation [24–26] or in terms of vaccine provision [27, 28], however none, to our knowledge, which cover infection risks comprehensively.

On the assumption that the standards applied in this audit were reasonable, particularly those developed as consensus guidelines, the overall compliance with most items to prevent infections was poor. It was clear that some specialties, for example Gastroenterology, performed better than others overall. However in this clinic, and indeed all the others, there were still a number of areas where significantly less than half of all patients received relevant interventions, such as screening for latent hepatitis B infection or hypogammaglobulinaemia. Aside from this, the suboptimal performance around three particular items was of most concern. First, for TB screening, less than half of all patients received a IGRA test, with the resulting lost opportunities to treat latent TB and prevent reactivation. Second, the failure to test for HIV in 44% of patients and hepatitis B [active or latent] in 24% of patients risked significant morbidity related to severe immunosuppression and late HIV diagnosis or reactivation of hepatitis. Third, the apparent failure to advise pneumococcal vaccination in over three quarters of patients and VZV or measles vaccines in most patients who were found to be non-immune put a significant proportion of patients at risk of these infections, which might have been avoided. UK vaccination guidelines recommend pneumococcal vaccination (PPV-23) to all adults over 65 years, as well as those undergoing immunosuppressive therapies, so not reinforcing this advice was regrettable. We suspect that the deficiencies in practice identified in this audit are not confined to our hospital and would expect many other health providers across the UK not to achieve the quality measures we set.

This audit has a number of limitations. Our definition of potent immunosuppression and the consequent need for PJP prophylaxis was arbitrary and may have overestimated risk of PJP in some groups, for example patients taking two less potent oral immunosuppressive drugs, or indeed underestimated the risk for haematology patients with underlying immunosuppression. This is one area where specialty guidelines are most different in terms of defining which drugs or combinations warrant prophylaxis, or in not mentioning PJP as a potential risk at all. One meta-analysis of clinical trials of PJP prophylaxis recommended using it where the risk of infection exceeded 3.5% [21], however there are significant challenges in establishing the risk associated with multiple combinations of immunosuppressive drugs in different diseases. Another limitation is that those reviewing medical records may not have noted certain tests done in other facilities including primary care outside our hospital, or assessment of immunity to VZV or measles which were not documented in records. Likewise, advice to have vaccines or other general advice may have been given however not written in records. Finally, the concept of applying a consensus guideline, for example the standard we used for determining whether PJP prophylaxis should be given, might well be regarded as unfair in specialties whose own guidelines did not use this measure. Hence if such consensus guidelines are felt to be appropriate for future audits in this area, specialties should be given the opportunity to adopt these recommendations in local guidelines prior to conducting audits.

Given the findings of this this audit, a number of interventions to improve practice should be considered to improve management of infection risks in patients undergoing immunosuppressive therapies. General education of specialist physicians around the risks of infections and how to mitigate these risks should be conducted through educational meetings and feedback of local audits in this area. Checklists have been used successfully in a number of areas of medicine to reduce patient safety risks [29], and would be ideal to be applied in patients prior to starting immunosuppressant drugs. Checklists could be designed to cover all 8 areas considered in this audit, and as was seen in one clinic in our audit using such a system, can be effectively implemented by a specialist nurse. Another intervention is to use order sets in electronic physician order entry systems [30]. Since it is possible to design such order sets in electronic systems, they would include BBV, VZV, measles, IGRA, immunoglobulin and chest x-ray tests, to enable simplification of the screening process. Some electronic prescribing systems also permit automatic co-prescriptions of cotrimoxazole, as PJP prophylaxis, with certain immunosuppressive drugs or chemotherapy. Finally, the use of a generic patient leaflet to provide information on screening tests, vaccines and prophylaxis requirements and other advice on infection risks would also reduce the amount of time required of healthcare professions to inform patients of such risks and the need for screening tests.

## Conclusions

This audit has shown that specialist clinics initiating immunosuppressive therapies are failing to comply with various guidelines to prevent infectious complications in most areas we audited. Cross-specialty, national guidelines to reduce infection risks, including areas not incorporated in specialist society guidelines, should be commissioned and interventions to improve compliance with guidelines are urgently needed to alleviate patient harm.

## Supplementary information

**Additional file 1: Table 1.** Consensus guideline for interventions or screening tests to prevent infections in patients undergoing immunosuppressive therapies, as part of audit.

## Data Availability

The datasets used and/or analysed during the current study are available from the corresponding author on reasonable request. Public access to the database used is closed. Administrative permission to use this dataset is not relevant as it was created by the research team.

## Abbreviations

BBV: Blood-borne viruses
BME: Black and minority ethnic
CMV: Cytomegalovirus
HBeAb: hepatitis B e antibody
HIV: Human immunodeficiency virus
IGRA: Interferon gamma release assay
PJP: *Pneumocystitis jiroveci* pneumonia
PPV-23: Pneumococcal polysaccharide vaccine – 23 valent
TB: Tuberculosis
VZV: *Varicella zoster* virus

## References

[1] Kumar D, Humar A, Plevneshi A (2008). Invasive pneumococcal disease in adult hematopoietic stem cell transplant recipients: a decade of prospective population-based surveillance. Bone Marrow Transplant.

[2] Issacs, D. Infectious risks associated with biologics. In N Curtis et al. Hot topics in infection and immunity in children IX. Advances in experimental medicine and biology, 764, 10.1007/978-1-4726-9_12. 10.1007/978-1-4614-4726-9_12 23654064

[3] Ford AC, Peyrin-Biroulet L (2013). Opportunistic infections with anti-tumor necrosis factor-α therapy in inflammatory bowel disease: meta-analysis of randomized controlled trials. Am J Gastroenterol.

[4] Kourbeti IS, Ziakas PD (2014). Biologic therapies in rheumatoid arthritis and the risk of opportunistic infections: a meta-analysis. Clin Infect Dis.

[5] Greendyke WG, Pereira MR (2016). Infectious complications and vaccinations in the post-transplant population. Med Clin North Am.

[6] Greenberg JD, Reed G, Kremer JM (2010). Association of methotrexate and tumour necrosis factor antagonists with risk of infectious outcomes including opportunistic infections in the CORRONA registry. Ann Rheum Dis.

[7] Patullo V (2016). Prevention of hepatitis B reactivation in the setting of immunosuppression. Clin Mol Hepatol.

[8] Rahier JF, Magro F, Abreu C (2014). Second European evidence-based consensus on the prevention, diagnosis and management of opportunistic infections in inflammatory bowel disease. J Crohns Colitis.

[9] Mowat C, Cole A, Windsor A (2011). Guidelines for the management of inflammatory bowel disease in adults. Gut.

[10] Baker R (2017). Post-operative Care in the Kidney Transplant Recipient.

[11] Chaganti S, Illidge T, Barrington S (2016). Guidelines for the management of diffuse large B-cell lymphoma. Br J Haematol.

[12] Warren RB, Weatherhead SC, Smith CH (2016). British Association of Dermatologists' guidelines for the safe and effective prescribing of methotrexate for skin disease 2016. Br J Dermatol.

[13] Smith CH, Jabbar-Lopez ZK, Yiu ZZ (2017). British Association of Dermatologists guidelines for biologic therapy for psoriasis 2017. Br J Dermatol.

[14] Heijstek M W, Ott de Bruin L M, Bijl M, Borrow R, van der Klis F, Koné-Paut I, Fasth A, Minden K, Ravelli A, Abinun M, Pileggi G S, Borte M, Wulffraat N M (2011). EULAR recommendations for vaccination in paediatric patients with rheumatic diseases. Annals of the Rheumatic Diseases.

[15] Smolen Josef S, Landewé Robert, Bijlsma Johannes, Burmester Gerd, Chatzidionysiou Katerina, Dougados Maxime, Nam Jackie, Ramiro Sofia, Voshaar Marieke, van Vollenhoven Ronald, Aletaha Daniel, Aringer Martin, Boers Maarten, Buckley Chris D, Buttgereit Frank, Bykerk Vivian, Cardiel Mario, Combe Bernard, Cutolo Maurizio, van Eijk-Hustings Yvonne, Emery Paul, Finckh Axel, Gabay Cem, Gomez-Reino Juan, Gossec Laure, Gottenberg Jacques-Eric, Hazes Johanna M W, Huizinga Tom, Jani Meghna, Karateev Dmitry, Kouloumas Marios, Kvien Tore, Li Zhanguo, Mariette Xavier, McInnes Iain, Mysler Eduardo, Nash Peter, Pavelka Karel, Poór Gyula, Richez Christophe, van Riel Piet, Rubbert-Roth Andrea, Saag Kenneth, da Silva Jose, Stamm Tanja, Takeuchi Tsutomu, Westhovens René, de Wit Maarten, van der Heijde Désirée (2017). EULAR recommendations for the management of rheumatoid arthritis with synthetic and biological disease-modifying antirheumatic drugs: 2016 update. Annals of the Rheumatic Diseases.

[16] British Society of Rheumatology. Guidelines section. https://www.rheumatology.org.uk/practice-quality/guidelines. Accessed 16 May 2020.

[17] Immunisation against infectious diseases. UK Government 2018. https://www.gov.uk/government/collections/immunisation-against-infectious-disease-the-green-book.

[18] UK National Institute for Health Care and Excellence. HIV testing: increasing uptake among people who may have undiagnosed HIV. 2016. https://www.nice.org.uk/guidance/ng60. 41428834

[19] UK National Institute for Health Care and Excellence. Hepatitis B and C testing: people at risk of infection. 2013. https://www.nice.org.uk/guidance/ph43.

[20] British HIV Association, British Association of Sexual Health and HIV, British Infection Society. UK National Guidelines for HIV Testing 2008. https://www.bhiva.org/file/RHNUJgIseDaML/GlinesHIVTest08.pdf.

[21] Green H, Paul M, Vidal L, Leibovici L (2007). Prophylaxis of *Pneumocystis* pneumonia in immunocompromised non-HIV-infected patients: systematic review and meta-analysis of randomized controlled trials. Mayo Clin Proc.

[22] Meiring J, de Silva TI, Snowden JA (2015). A study of adherence to a vaccination schedule following adult allogeneic haematopoietic stem cell transplants in UK transplant centre. Bone Marrow Transplant.

[23] Miller PDE, da Silber I, Skinner R (2017). Routine vaccination practice after adult and paediatric allogeneic haematopoietic stem cell transplant: a survey of UK NHS programmes. Bone Marrow Transplant.

[24] Marty FM, Rubin RH (2006). The prevention of infection post-transplant: the role of prophylaxis, preemptive and empiric therapy. Transpl Int.

[25] Fischer SA, Avery RK (2009). Screening of donor and recipient prior to solid organ transplantation. Am J Transplant.

[26] Working party of the British Transplant Society. British Guidelines for Hepatitis B & Solid Organ Transplantation. 2018. https://bts.org.uk/wp-content/uploads/2018/03/BTS_HepB_Guidelines_FINAL_09.03.18.pdf.

[27] Cordonnier C, Ljungman P, Juergens C (2015). Immunogenicity, safety, and tolerability of 13-valent pneumococcal conjugate vaccine followed by 23-valent pneumococcal polysaccharide vaccine in recipients of allogeneic hematopoietic stem cell transplant aged >/= 2 years: an open-label study. Clin Infect Dis.

[28] Rubin LG, Levin MJ, Ljungman P (2014). 2013 IDSA clinical practice guideline for vaccination of the immunocompromised host. Clin Infect Dis.

[29] Clay-Williams R, Colligan L (2015). Back to basics: checklists in aviation and healthcare. BMJ Qual Safety.

[30] Li RC, Wang JK, Sharp C, et al. When order sets do not align with clinician workflow: assessing practice patterns in the electronic health record. BMJ Qual Saf Published Online First: 04 June 2019. 10.1136/bmjqs-2018-008968. 10.1136/bmjqs-2018-008968 PMC6868292 31164486

