# Supplementary material for: Adherence to guidelines across different specialties to prevent infections in patients undergoing immunosuppressive therapies
Source: BMC Infect Dis. 2020 May 20;20:359. doi: 10.1186/s12879-020-05082-8 (PMC7238578; doi:10.1186/s12879-020-05082-8)
Supplement: Supplementary file 1 — Additional file 1: Table 1. Consensus guideline for interventions or screening tests to prevent infections in patients undergoing immunosuppressive therapies, as part of audit. [file 12879_2020_5082_MOESM1_ESM.doc]

**Supplementary Table 1. Consensus guideline for interventions or screening tests to prevent infections in patients undergoing immunosuppressive therapies, as part of audit.**

| **Intervention or Test** | **Definition of intervention/test** |
| --- | --- |
| General advice to patients on risk of infection and live vaccines on therapy | Discussion documented in case notes |
| Screening for active or latent tuberculosis (TB) | Pre-therapy chest x-ray and clinical evaluation for active TB  Interferon-γ reactivation (IGRA) test |
| Screening for current or previous blood-borne virus infections | HIV, hepatitis B (HBsAg & HBcAb) and hepatitis C (HCV IgG) tests |
| Screening for immunity to vaccine-preventable infections | HBsAb/HBcAb, Measles IgG, VZV IgG tests |
| Recommendation of vaccination with hepatitis B, measles or VZV | For patients with either tests suggesting lack of immunity or no history of previous infection |
| Screening for hypogammaglobulinaemia | Total immunoglobulins test |
| Recommendation of vaccination with influenza and pneumococcal vaccine | For all patients: annual influenza vaccinations and one-off pneumococcal (PPV-23) vaccine if not previously received |
| *Pneumocystis jiroveci* pneumonia (PJP) prophylaxis | Provision of cotrimoxazole (or alternative) to patients defined as undergoing more potent immunosuppression |

HBsAg – hepatitis B surface antigen; HBsAb – hepatitis B surface antibody; HBcAb – hepatitis B surface antibody; VZV – varicella zoster virus; PPV-23 – pneumococcal polysaccharide 23-valent vaccine
